# Poly(lactic acid-*co*-oxacyclohexadecenlactone) (PLH): A Bio-Based Substrate for Flexible Printed Electronic Devices

**DOI:** 10.1021/acsami.6c04927

**Published:** 2026-06-03

**Authors:** Aida Visús Martínez, Nathalie Marcela Cerón, José L. Monzón, Miquel Redón, David Sánchez, Jordi Sacristán, Xavier Jordà, Carlos Domínguez Horna, Xavier Muñoz Berbel

**Affiliations:** † 120392 Institute of Microelectronics of Barcelona (IMB-CNM), Universitat Autònoma de Barcelona, Campus UAB, 08193 Bellaterra, Spain; ‡ Artificial Nature S.L., Carrer de Baldiri Reixac 10, 08028 Barcelona, Spain

**Keywords:** printed electronics, flexible substrates, green electronics, biobased polymers, surface properties

## Abstract

The increasing demand for sustainable materials in flexible printed electronics has driven interest in biobased substrates as alternatives to petroleum-derived polymers such as polyethylene terephthalate (PET). This study evaluates a poly­(lactic acid-*co*-oxacyclohexadecenlactone) (PLH) polymer as a biobased substrate for flexible electronics. Mechanically, PLH exhibited a lower Young’s modulus (26 ± 3 MPa) than PET (110 ± 10 MPa), indicating greater flexibility, and demonstrates excellent elastic recovery, with no permanent deformation observed after a 0.5 mm extension (2 cm × 0.8 cm samples). Dielectric strength tests confirmed both substrates withstand voltages exceeding 5 kV, with no variation after curing at 60 °C. Thermogravimetric analysis (TGA) of the PLH reveals a temperature at 5% mass loss (T_5_%) of approximately 273 °C, indicating the onset of thermal degradation. The corresponding DTG curve shows a main degradation peak at around 295 °C, associated with the primary decomposition process, confirming sufficient thermal stability for printing processes using organic and aqueous inks. Contact angle measurements indicate moderate wettability (PLH: 80° ± 4; PET: 75° ± 5), while confocal microscopy reveals a slightly rougher topside for PLH compared to the smoother PET surface. Nevertheless, PLH supports reliable screen printing of conductive tracks (0.6 cm × 100 μm) with adhesion meeting ASTM D3359 standards. The functional performance of PLH, combined with its biobased origin and validated environmental assessment, highlights its potential as a substrate for flexible electronics with moderate thermal requirements. Future work should address bending durability and surface homogeneity to further enhance PLH applicability.

## Graphical abstract

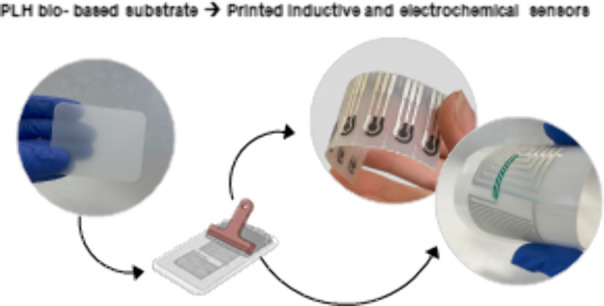

## Introduction

Printed electronics have emerged as a transformative technology for the development of low-cost, lightweight, and mechanically flexible devices, enabling applications such as wearable sensors, rollable displays, and disposable diagnostic tools. This field relies on additive manufacturing techniques, including screen printing and inkjet printing, which enable scalable and cost-effective fabrication of electronic components on a wide range of substrates.^1,2^ Compared with conventional microfabrication approaches, printed electronics offer reduced material consumption, lower energy requirements, and improved compatibility with flexible substrates, making them particularly attractive for next-generation conformal and wearable electronic systems.^3,4^

Despite these advances, the widespread adoption of printed electronics remains constrained by the environmental impact of commonly used substrate materials. Flexible polymeric substrates such as polyethylene terephthalate (PET) and polyethylene naphthalate (PEN)^5^ are derived from fossil resources and are inherently nonbiodegradable, posing significant sustainability challenges, particularly for short-lifecycle or disposable devices.^6^ These concerns are amplified by the rapid growth of electronic waste (e-waste), which reached approximately 62 million tonnes globally in 2022 and is projected to increase to 82 million tonnes by 2030.^7,8^ Although PET is widely recyclable in high-volume applications, its practical recyclability in flexible printed electronics is limited by multimaterial architectures (e.g., conductive inks, metallic tracks, adhesives, and encapsulation layers), which hinder compatibility with conventional recycling streams and complicate polymer recovery.^3,9–11^

Beyond macroscopic waste accumulation, conventional polymers also contribute to micro- and nanoplastics pollution.^8^ These particles, generated through environmental degradation, are persistent, potentially bioaccumulative,^9^ and capable of transporting hazardous substances.^12^ In the context of printed electronics, particularly disposable devices, the reliance on nondegradable substrates may exacerbate this issue, reinforcing the need for alternative materials aligned with green electronics and circular economy principles.^13,14^

To address these challenges, increasing attention has been devoted to biodegradable organic materials, encompassing both natural and synthetic polymers.^14^ Substrates derived from natural sources, such as cellulose,^15^ silk,^16^ starch,^10^ or chitosan^11^ offer inherent biodegradability and originate from renewable feedstocks, rendering them attractive for environmentally benign electronic applications.^14,17^ However, their implementation in printed electronics is often hindered by limited thermal tolerance, sensitivity to humidity, dimensional instability during processing, and incompatibility with industrially relevant fabrication techniques such as screen printing and inkjet deposition.^18^ Beyond natural polymers, several synthetic biodegradable polymers, including poly­(lactic-*co*-glycolic acid) (PLGA), polycaprolactone (PCL), and poly­(1,8-octanediol-*co*-citrate) (POC), have been investigated as potential flexible substrates. These materials enable transient and bioresorbable electronic platforms designed to degrade under aqueous or physiological conditions.^19^ Despite these advantages, their mechanical integrity, thermal stability, and dimensional robustness under typical printing and postprocessing conditions often remain insufficient for large-area manufacturing, multilayer device integration, and scalable screen-printing workflows.^17,18^

To date, relatively few studies have adopted a comprehensive, multiparametric approach to evaluating biodegradable substrates for printed electronics. Critical interdependencies between surface morphology, electrical insulation, thermal stability, and mechanical performance are frequently overlooked, despite their central importance in determining material suitability under realistic processing and operational conditions. Consequently, a clear materials gap persists for substrates capable of simultaneously delivering mechanical flexibility, printing compatibility, thermal robustness and end-of-life biodegradability, a balance essential for next-generation sustainable electronic systems. Recent advances in functional and interfacial materials have further highlighted the importance of scalable fabrication strategies and interface engineering in enabling next-generation electronic devices.^20,21^ In particular, the development of advanced material systems through scalable processing routes underscores the need to simultaneously address material compatibility, mechanical robustness, and integration potential for practical device implementation.

In this context, the present work reports an in-depth characterization of PLH (i.e., Poly­(lactic acid-*co*-oxacyclohexadecenlactone), a novel synthetic biobased copolyester developed and patented by Artificial Nature S.L. (PCT/WO2023/07880; EP4426769 B1). PLH is synthesized from renewable feedstocks such as sugar cane and corn stover, and is designed for controlled biodegradation. The polymer belongs to a new class of biobased materials designed following green chemistry principles, minimizing the use of hazardous solvents and reducing energy consumption during synthesis. Importantly, PLH production is compatible with conventional polymer-processing techniques and infrastructure. The environmental performance of PLH has been independently assessed through a third-party validation under the EU “Do No Significant Harm” (DNSH) framework, which evaluates the absence of significant harm across multiple environmental objectives. Although a full life cycle assessment (LCA) is not yet available, this validation supports the plausibility of its environmental profile.

This study evaluates the suitability of PLH-based films as substrates for flexible printed electronics through a comprehensive analysis of their morphology, electrical properties, thermal stability and mechanical robustness. The compatibility of PLH with screen-printed conductive inks and its performance under bending stress are also investigated. All results are benchmarked against commercial PET films, which serve as the industry standard for flexible printed electronics.

By integrating sustainable material design with the functional requirements of printed electronics, PLH offers a viable biobased alternative to conventional fossil-derived polymer substrates, contributing to the development of more environmentally responsible flexible electronic systems.

## Materials and Methods

### Reagents and Materials

The films made of PLH were provided by Artificial Nature S.L. – DAN*NA (Barcelona, Spain). The synthesis and structural characterization of Poly­(lactic acid-*co*-oxacyclohexadecenlactone) (PLH) have been previously reported.^22^ Polyethylene terephthalate (PET) substrates (STS A.00-A.00, Cyclops Synergies, Spain) were used as reference material. Silver/Silver Chloride (Ag/AgCl: 60/40) ink (C2130809D5, SunChemical, supplied by IMCD, Spain), Silver ink (LOCTITE ECI 1010, Henkel adhesives, supplied by Cyclops Synergies, Spain) and carbon-graphite ink (C2030519P4, Sun Chemicals, supplied by IMCD, Spain) were used for the development of different prototypes by screen printing technique. Additionally, a UV-curable dielectric ink (D2121102P1, Sun Chemicals, supplied by IMCD, Spain) was screen-printed and cured using a UV flood lamp (Model 5000 Flood, Dymax, USA) operated with a 400-W power supply (Dymax, USA). Potassium chloride (KCl, 99.0–100.5%, P3911, Sigma-Aldrich, Darmstadt, Germany), potassium ferricyanide (K_3_[Fe­(CN)_6_], 60299, Sigma-Aldrich, Darmstadt, Germany), and potassium ferrocyanide trihydrate (K_4_[Fe­(CN)_6_]·3H_2_O, P3289, Sigma-Aldrich, Darmstadt, Germany) were used for sensor characterization.

### Surface and Morphological Characterization

Surface wettability was assessed through contact angle measurements using a KRÜSS DSA30E instrument (KRÜSS GmbH, Hamburg, Germany). Surface topography, and surface roughness were characterized using a PLμ NEOX 3D optical confocal profilometer (Sensofar, Spain). The surface roughness was quantified by the root-mean-square roughness (Sq). Additionally, a Micromeritics ASAP 2020 analyzer (Micromeritics, USA) was used to determine the size and volume distribution of mesopores of the PLA and PET samples by nitrogen adsorption at 77 K according to Barrett–Joyner–Halenda (BJH) model. The thicknesses of the films were determined using a Mitutoyo digital caliper (245–5676, RS, Spain). Measurements were performed at multiple positions (n = 3) across each sample to account for potential thickness variations, ensuring a representative average value for subsequent electrical and mechanical analyses.

### Insulating Characterization

The insulating properties of the substrate were evaluated by measuring its dielectric breakdown voltage and electrical capacitance. Dielectric breakdown voltage measurements were conducted using a Keithley 2400 SourceMeter (Keithley Instruments, USA) coupled with an EuroTest HPP-120–256-IEEE high-voltage tester (EuroTest, Spain) reaching voltages of up to 5.5 kV. The relative electrical permittivity (ε_r_, also known as the dielectric constant) was assessed by measuring capacitance through electrochemical impedance spectroscopy (EIS). Measurements were carried out with a MultiPalmSens4 multichannel potentiostat/galvanostat (PalmSens, The Netherlands) in a parallel-plate ’sandwich’ configuration, where the substrates were positioned between 0.6 cm diameter aluminum electrodes (AT506 adhesive tape, United Kingdom) to ensure a stable and well-defined contact area. The local film thickness beneath the electrodes was measured prior to each dielectric experiment and incorporated into the εr calculation to correct for possible variations. The frequency window from 0.1 to 10^6^ Hz was selected to capture both low-frequency interfacial polarization and the intrinsic dipolar response of the dielectric layer. A DC bias of 0.2 V was applied together with a 100 mV AC perturbation, which provided a clearer and more stable impedance response across the full frequency range while preserving linear dielectric behavior.

The dielectric constant was calculated using the parallel-plate capacitor model (eq 1), where *C* represents the capacitance measured at 10 kHz, *d* corresponds to the dielectric thickness of the dielectric layer, *A* denotes the effective electrode area, and ε_0_ = 8.854 × 10^–12^ F/m is the permittivity of free space.

$$\varepsilon_{r}=\frac{C\cdot d}{\varepsilon_{0}\cdot A}$$ 1

### Thermal Characterization

Thermogravimetric analysis (TGA) was used to evaluate the thermal stability and degradation profile of the PLH copolyester and PET. Measurements were conducted using a Discovery TGA 550 thermogravimetric analyzer (TA Instruments, USA) under a nitrogen atmosphere from 30 to 800 °C at a constant heating rate of 10 °C/min. Both the mass-loss curve (TGA) and its derivative thermogravimetry (DTG) were recorded to identify the temperature corresponding to the onset of decomposition, the temperature at fixed mass-loss percentages (T_5_%), and the maximum degradation rate (Tmax). This methodology allows distinguishing early degradation events from the main decomposition step and enables direct comparison with the thermal behavior of PET and other reference materials.

### Mechanical Characterization

Young’s modulus was determined by tensile testing of rectangular samples (2 × 0.8 cm, n = 3), using a custom setup combining a Zaber X-MCB1 motor controller (Zaber Technologies, Canada) with a force sensor (MR03–20, Mark-10 Corporation, USA). Measurements were performed at a constant speed of 1 mm/min and a maximum extension of 0.5 mm. The modulus was calculated from the linear region of the stress–strain curve, considering the slope of the elastic response of the material, as defined in eq 2, where *F* is the applied force, *A* is the sample’s cross-sectional area, L_0_ is the initial gauge length, and ΔL is the corresponding extension within the elastic region.

A detailed schematic of the experimental setup and the sample preparation procedure is provided in Figure S1 of the Supporting Information.

$$E=\frac{F\cdot L_{0}}{A\cdot \Delta L}$$ 2

### Printing Process and Ink Adhesion

Printed conductive traces were fabricated using a manual screen-printing machine (Paymser, Spain) equipped with 100 threads/cm mesh screens oriented at 45° (Paymser, Spain). The design of the masks was made with CorelDRAW 2020 software (Ottawa, Canada). Ag/AgCl conductive lines with dimensions of 0.6 cm in length and 100 μm in width were printed at room temperature and subsequently cured at 60 °C during 30 min. To evaluate electrical integrity under dimensional deformation due to the expansion-contraction of the substrate, the resistance of these lines was measured with the Fluke 77 IV digital multimeter (AMIDATA, Spain). The Adhesion of the printed ink to the substrates was evaluated using a peel test with Scotch Crystal Tape 600 (3M, 498–0887, RS, Spain) exhibiting a nominal peel adhesion value of ∼20 N/25 mm (manufacturer specification). The tape was applied firmly over the printed area and peeled off at a 180° angle, following a semiquantitative adhesion assessment based on the general guidelines of ASTM D3359. Ink removal on the tape and on the substrate was visually inspected to evaluate adhesion performance.

### Characterization of Printed Sensors

The inductive sensor measurements were carried out using an impedance analysis module (Agilent 4294A, Agilent Technologies, USA). Electrochemical measurements were performed using a three-electrode printed system comprising a carbon working electrode (WE), a carbon counter electrode (CE), and an Ag/AgCl reference electrode (RE). All experiments were conducted at room temperature using a MultiPalmSens4 potentiostat/galvanostat (PalmSens, The Netherlands).

## Results and Discussion

### Surface and Morphological Properties

Thickness measurements were performed using a digital caliper. The fabrication process yielded in a semitransparent, smooth, and macroscopically uniform PLH films (Figure 1A) with an average thickness of 279 ± 12 μm, measured on rectangular samples (2.0 × 0.8 cm, N = 17). Thickness measurements revealed a moderate thickness variation across the substrate, with values ranging from 260 to 300 μm; however, no visible defects or cracks were observed, indicating good macroscopic continuity of the films.

**Figure 1:**
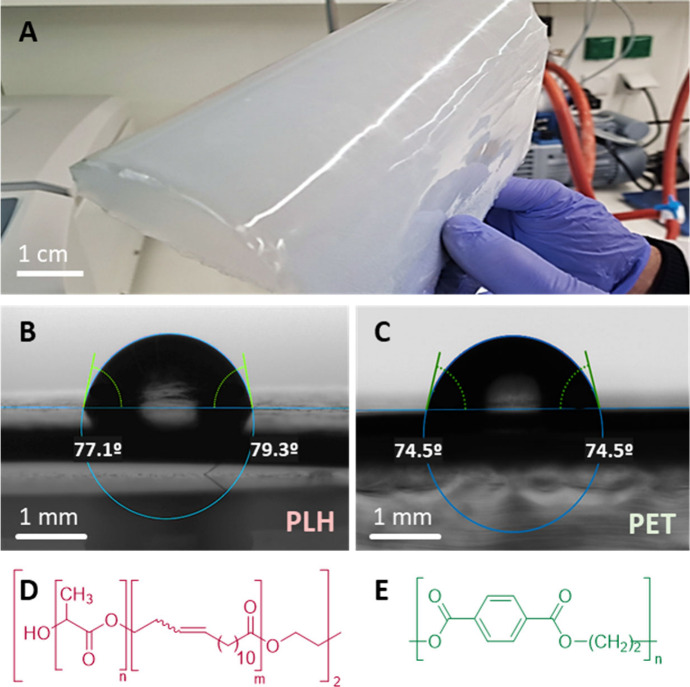
Morphology, wettability and chemical structures of PLH and PET. (A) PLH film showing a smooth, slightly transparent surface. (B) Contact angle measurements of PLH and (C) PET. Chemical structures of the repeating units of PLH (D) and PET (E).

Surface wettability was evaluated by static contact angle measurements using deionized water as probe liquid. As shown in Figure 1B, PLH exhibited a water contact angle of 80 ± 4°, corresponding to moderate wettability. This value is comparable to that of commercial PET substrates (Figure 1C), which displayed a slightly lower contact angle of 75 ± 5°. Despite the chemical differences between the two polymers, both contain ester functional groups that introduce some polarity, while also incorporating hydrophobic segments – long aliphatic chains in PLH (Figure 1D) and aromatic rings in PET (Figure 1E). As a result, their macroscopic wettability remains similar, suggesting comparable surface energy characteristics favorable for uniform ink spreading and adhesion in screen printing processes.^23^ Such wettability profiles are generally considered optimal for conductive ink deposition, promoting continuous track formation without excessive spreading or dewetting.

Representative lateral (side-view) macroscopic images of PET and PLH films are presented in Figure 2A. No significant differences in bending response are observed between the two substrates at this scale. Surface topography and roughness were assessed by confocal microscopy. Representative images PET (Figure 2B) reveal a highly smooth and uniform surface, consistent with its industrial extrusion-based manufacturing process. In contrast, of PLH films (Figure 2C) exhibit a moderately rougher surface, with noticeable differences between the topside (air-exposed surface) and the underside (mold-contacted surface). The topside of PLH shows relatively homogeneous texture, whereas the underside presents noticeable surface texture differences and increased morphological features. These differences are attributed to the solvent-casting process used to fabricate PLH films. Solvent casting is known to induce surface inhomogeneities due to solvent evaporation dynamics, polymer chain rearrangement, and interfacial effects at the air and mold interfaces. As a result, the mold-contacted underside typically exhibits higher roughness and less uniform morphology than the air-exposed topside. By contrast, industrial polymer films such as PET are commonly produced by melt extrusion, a process that yields smoother and more uniform surfaces. In the present study, all printing experiments were intentionally conducted on the PLH topside, where surface uniformity and wettability were more suitable for printed electronic applications.

**Figure 2:**
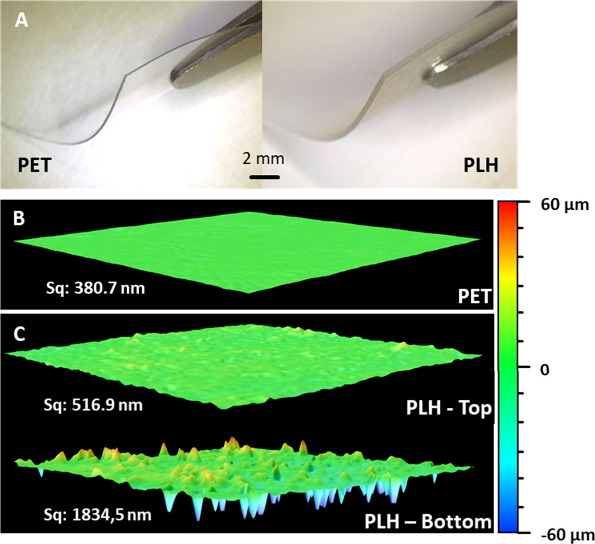
Morphology of substrates. (A) Cross-sectional photographs of PET and PLH substrates (B) Three-dimensional confocal surface height map of PET acquired over an area of 477.25 × 477.25 μm^2^, with the corresponding root-mean-square roughness (Sq). (C) Three-dimensional confocal surface height maps of the top and bottom sides of PLH acquired over the same area, together with the corresponding Sq values.

To complement the surface morphology analysis, nitrogen adsorption measurements were performed to assess the mesostructural characteristics of the substrate. The average pore diameter was calculated using the relation *4 V/A*, where *V* is the cumulative pore volume and *A* is the specific surface area obtained from the BJH model. The PLH substrate exhibited an average pore diameter of 4.25 nm, while PET showed a slightly larger value of 4.40 nm. Both values fall within mesopore range (approximately 4–5 nm), indicating comparable nanoscale surface textural features for the two materials. No significant differences in average pore diameter were observed, suggesting that mesoporous surface features are unlikely to be a differentiating factor influencing ink adhesion or print quality under the conditions investigated.

Overall, PLH and PET exhibited comparable wettability and mesoporosity, while confocal microscopy revealed differences in surface roughness associated with their respective fabrication processes. PET presented a smoother and more uniform surface, whereas PLH showed moderate roughness with distinct topside and underside morphologies. These surface characteristics provide a basis for correlating substrate morphology with printing behavior and electrical performance, which are examined in the subsequent sections.

### Insulating Properties

The insulating performance of PLH was evaluated through impedance measurements across different frequencies. As expected for nonconductive polymer films, the impedance magnitude increased at lower frequencies due to the capacitive nature of the material (|Z| ∝ 1/f). This frequency-dependent behavior is consistent with a dielectric polymer and reflects a response dominated by polarization processes rather than charge transport.^24^ From these measurements, it is possible to determine the relative permittivity (ε_r_), which describes the ability of a material to store electrical energy under an applied electric field and is a critical parameter for insulating substrates, as lower dielectric constants are typically associated with reduced dielectric losses and lower parasitic capacitances.

The ε_r_ of PLH was measured at 10 kHz, yielding a value of 3.7 ± 0.9. This frequency was selected because the capacitance values were highly stable and reproducible at 10 kHz, whereas measurements at lower frequencies exhibited increased noise and signal fluctuations due to interfacial polarization effects. The permittivity values were calculated using a parallel-plate capacitor model, considering the measured thickness of each sample. It should be noted that these values represent apparent permittivity under the specific geometrical and frequency conditions of the measurement. This value falls within the typical range reported for engineering thermoplastics and is comparable to that of PET (∼3).^25^ These results indicate that PLH exhibits adequate insulating behavior for flexible electronic substrates operating at kilohertz frequency ranges.

The dielectric strength of PLH was subsequently assessed and compared with that of commercial PET substrates using the experimental configuration shown in Figure 3A. In this setup, an increasing voltage was applied across the samples until electrical breakdown occurred. The corresponding current–voltage profiles for PLH and PET (Figure 3B) show a stable voltage increase prior to breakdown. Both materials withstood applied voltages exceeding 5 kV, demonstrating robust insulating performance and comparable breakdown behavior.^26^ To evaluate the influence of thermal processing, PLH and PET films were subjected to curing at 60 °C for different durations (0, 15, 30, and 60 min). No significant changes in dielectric strength were observed as a function of curing time for either material. Additionally, no macroscopic deformation or visible surface defects were observed. These results indicate that moderate thermal curing does not adversely affect the insulating integrity of either substrate under the conditions investigated, supporting the suitability of PLH as an alternative substrate to PET.^14^

**Figure 3:**
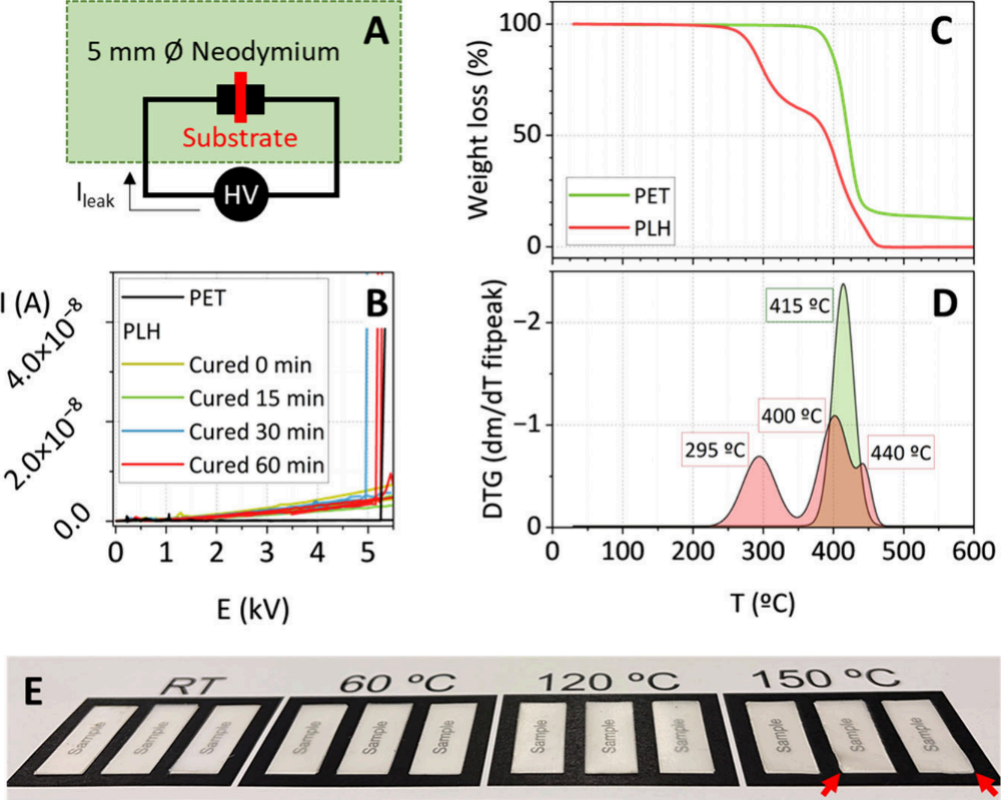
Electrical and thermal stability. (A) Schematic of the electrical circuit used for dielectric breakdown testing of PLH and PET substrates, designed to apply a uniform electric field and measure breakdown voltages. (B) Current–Voltage profiles for PET and PLH substrates, illustrating the increase in applied potential until dielectric breakdown at voltages exceeding 5 kV. (C) TGA curve of PET and PLH polymers under nitrogen atmosphere, showing percentage mass loss as a function of temperature. (D) DTG curve derived from the TGA data in, illustrating the rate of mass loss. (E) Photographs of PLH specimens after thermal treatment at room temperature (RT), 60 °C, 120 °C, and 150 °C for 15 min. The images illustrate the macroscopic dimensional changes of the substrates after heating, allowing visual assessment of thermal expansion or contraction, with permanent deformations observed at 150 °C (highlighted by red arrows).

### Thermal Properties

The thermal degradation behavior of PLH and PET was evaluated by TGA together with derivative thermogravimetry (DTG), as shown in Figure 3C–D. Both materials exhibit distinct mass-loss profiles associated with their polymeric structures. PLH displays a gradual initial mass loss with a temperature at 5% mass loss (T_5_%) of approximately 273 °C, marking the onset of thermal degradation. The DTG curve reveals multiple degradation steps, with a first pronounced peak at 295 °C followed by a second degradation event centered near 400 °C, and a less intense feature at higher temperatures (440 °C). This multistep degradation behavior was consistent with the block copolymer structure of PLH, where each segment contributed independently to the overall thermal response. The presence of distinct DTG peaks could therefore be attributed to the sequential degradation of chemically different blocks within the copolymer. PLH underwent complete mass loss during TGA, reaching negligible residual mass at 600 °C, consistent with its fully organic composition and the absence of inorganic fillers or char-forming components.

In contrast, PET displays a markedly different degradation profile. From TGA curve, the T_5_ % is ∼380 °C, indicating a substantially higher onset of degradation compared with PLH. The DTG trace further reveals a single sharp degradation peak centered at ∼420 °C, in agreement with reported values in the literature.^27^ The higher *T*_max_ and the absence of secondary peaks indicate a more uniform chain-scission process governed by the thermal decomposition of aromatic terephthalate units. PET retains approximately 10% residual mass at 600 °C, likely associated with thermally stable char formation or the presence of inorganic additives.^28^ This contrast with the complete volatilization observed for PLH and highlights the distinct end-of-life thermal behavior of the two polymers.

From a processing standpoint, the lower T_5_ % and *T*_max_ of PLH compared to PET indicate reduced intrinsic thermal stability, which is expected considering its highly labile aliphatic segments. Nevertheless, the thermal stability of PLH remains fully compatible with manufacturing processes for digital and printed electronics, as typical curing temperatures for conductive and functional inks remain below 150 °C. Thus, despite its lower degradation threshold relative to PET, PLH can be safely processed under standard industrial conditions without risk of thermal degradation.

Following the thermal characterization, the dimensional stability of PLH was evaluated to assess its behavior under processing-relevant temperatures for printed electronics applications. Rectangular PLH specimens (0.8 × 2.0 cm) were subjected to isothermal treatments at room temperature (RT), 60 °C, 120 °C, and 150 °C for 15 min. After heating, the samples were placed in a simple paper template corresponding to their initial dimensions, as shown in Figure 3E, allowing direct observation of thermally induced expansion or contraction. This study was not performed for PET, given its significantly higher thermal stability range as evidenced by TGA.

At 60 °C, PLH did not exhibit any apparent dimensional change or deformation, retaining its original shape and rigidity after cooling. In contrast, samples treated at 120 °C became slightly more malleable immediately after removal from the oven; however, upon cooling and drying at room temperature (within a few seconds), the substrate largely recovered its initial rigidity without permanent deformation. These results indicate that PLH is a suitable candidate for printing processes involving heat-curing inks at temperatures up to 120 °C.

When the temperature was increased to 150 °C, slight but measurable deformation of the material was observed, leading to permanent dimensional distortions after cooling. These deformations, highlighted by red arrows in Figure 3E, indicate the onset of macroscopic thermal instability under exposure to elevated temperatures. Overall, this analysis confirms that PLH maintains adequate dimensional stability up to 120 °C, supporting its suitability for low-temperature processing applications such as printed and digital electronics.

These results were used to define the thermal conditions selected for the subsequent mechanical characterization, including samples processed at 150 °C to assess the effect of thermally induced deformation.

### Mechanical Properties

The mechanical behavior of the PLH substrate was evaluated by uniaxial tensile testing to determine its Young’s modulus, using commercial PET films as a reference material. Tensile measurements were performed by applying a controlled extension of 0.5 mm, corresponding to the linear elastic region of stress–strain response, from which Young’s modulus was extracted. Young’s modulus provides a quantitative measure of substrate stiffness and is a key parameter for flexible electronic applications, where mechanical compliance is required to accommodate bending and deformation. A detailed description of the experimental setup and measurement procedure is provided in the Supporting Information (Figure S1).

As summarized in Table 1, PET exhibited a Young’s modulus of 110 ± 10 MPa, while PLH showed significantly lower values (25–27 MPa). No statistically significant differences were observed in the Young’s modulus of PLH as a function of curing temperature within the experimental error, indicating a stable mechanical response across the investigated thermal processing range, despite slight deformation observed at 150 °C. This reduced stiffness is advantageous since enabling better adaptation of the substrate to mechanical deformation without generating excessive stress in the printed functional layers.^23^

**Table 1:** Young’s Modulus of PET and PLH Substrates under Different Curing Conditions (N = 3)

**Material**	**Young’s modulus**(MPa)
PET	110 ± 10
PLH - RT	26 ± 5
PLH - 60 °C - 15 min	25 ± 2
PLH - 120 °C - 15 min	27 ± 3
PLH - 150 °C - 15 min	25 ± 2

Elastic recovery was further evaluated by unloading the samples after the extension. Upon release of the applied strain, no measurable permanent deformation was observed within the tested strain range, indicating excellent elastic recovery. These results suggest that, despite its lower stiffness, PLH maintains dimensional stability under mechanical loading. Further investigation of cyclic bending durability will be required to assess long-term mechanical reliability in flexible and wearable electronic applications.

### Printing and Ink Adhesion

The compatibility of PLH with printed electronics fabrication was evaluated through the electrical and mechanical characterization of conductive tracks deposited on PET and PLH substrates. Conductive lines with a nominal width of 100 μm and a length of 0.6 cm were fabricated using screen-printing process (Figure 4A), and their electrical resistance was measured using a digital multimeter. Printed tracks on both PET and PLH substrates exhibited low resistance, with average values of 2.00 and 1.50 mΩ respectively, indicating good electrical continuity.

**Figure 4:**
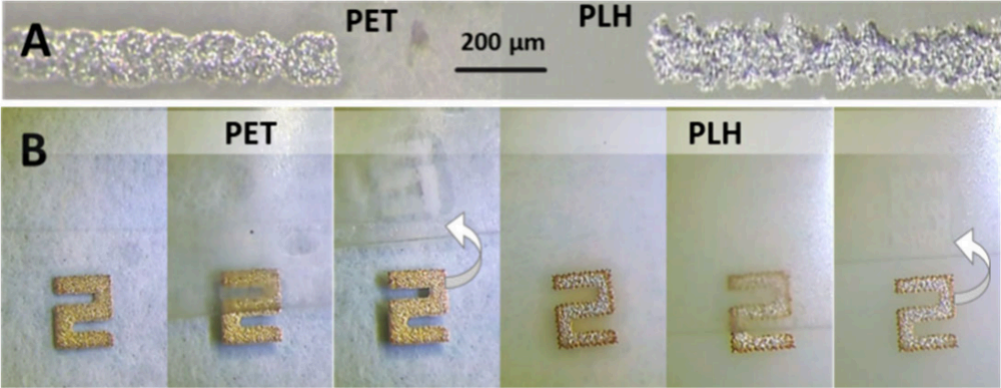
Screen-printed Ag/AgCl Printing of commercial inks on substrate. (A) Ag/AgCl conductive tracks with a nominal width of 100 μm. (B) Tape test procedure before tape application, during tape removal, and after detachment for both materials.

Adhesion of the printed patterns was evaluated using the ASTM D3359 tape test (Method B) with a standardized transparent pressure-sensitive adhesive tape. Figure 4B shows the tape test procedure, including the printed design before tape application, during tape removal, and the final state of the design after transfer, for both materials. Printed patterns exhibited good adhesion to the substrate, showing only minor and localized coating removal after tape detachment, which corresponds to a 4B classification according to the ASTM standard, indicating good adhesion, with less than 5% coating removal after tape detachment.

### Inductive Sensor

Following the comprehensive characterization of the mechanical, electrical, and surface properties of the PLH substrate, a proof-of-concept inductive sensor was developed to assess the suitability of this material for functional sensing applications. The objective of this section is not to optimize sensor performance, but rather to demonstrate that the PLH substrate can support the fabrication and operation of an active inductive device under realistic conditions. To this end, a simple planar coil was printed directly onto the PLH substrate, forming a compact inductive sensing element (Figure 5A). The coil geometry was selected to ensure stable electrical behavior over a wide frequency range while maintaining compatibility with printed electronics processes and preserving the mechanical flexibility of the substrate. This straightforward design enables direct exposure of the sensing element to the surrounding medium, allowing the inductive response to be modulated by the electromagnetic properties of materials in contact with the coil.

**Figure 5:**
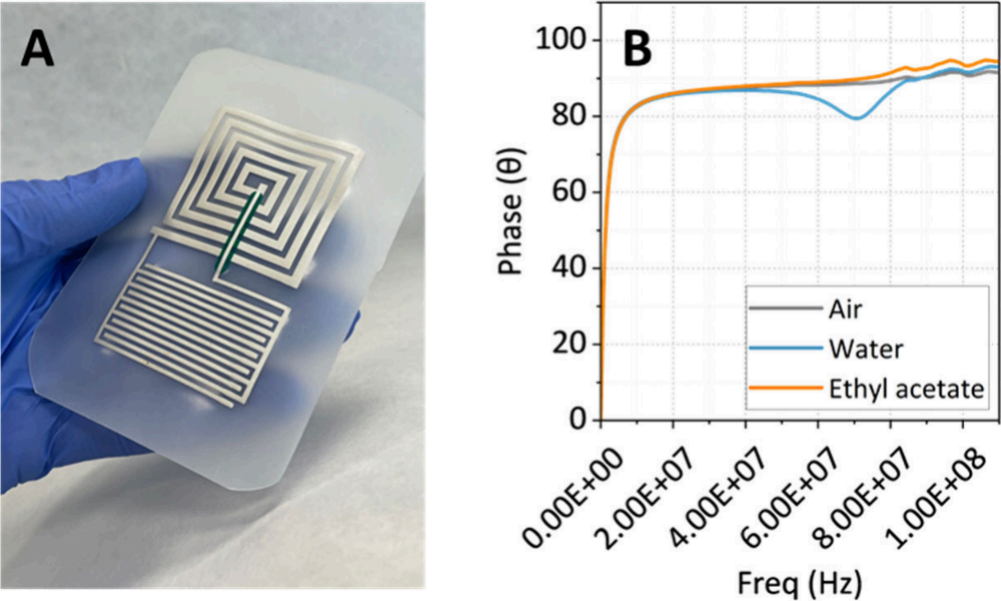
Development of an inductive sensor. (A) Flexible printed device integrating a spiral inductor and an interdigitated electrode on an PLH substrate. (B) Phase response as a function of frequency for three reference media—air, water, and ethyl acetate—demonstrating the distinct dielectric signatures and sensitivity of the sensor across the radio frequency range.

The functionality of the prototype arises from the interaction between the alternating magnetic field generated by the coil and the liquid medium in direct contact with its surface. Variations in the liquid’s electromagnetic properties, such as permittivity, can be detected in the impedance response of the coil. Impedance spectroscopy measurements (Figure 5B) confirmed that both the magnitude and, more distinctly, the phase of the impedance vary depending on the liquid present.

Distinct and reproducible phase shifts were observed when the coil was exposed to air, water, and ethyl acetate across the investigated frequency range from 0.1 to 10^8^ Hz. The three media were intentionally selected as extremes in dielectric behavior:^29^ water is a highly polar liquid with a high relative permittivity (ε_r_ ∼ 80), ethyl acetate is a weakly polar organic solvent (ε_r_ ∼ 6), and air, effectively nonpolar (ε_r_ ∼ 1). These phase differences confirm that the printed coil remains electrically functional and responsive to changes in the surrounding medium under the tested conditions.

### Electrochemical Sensor

A flexible three-electrode electrochemical sensor was fabricated on the PLH substrate using screen printing (Figure 6A). The device consisted of silver (Ag) tracks, circular carbon working electrode (WE), a carbon counter electrode (CE) and a silver/silver chloride (Ag/AgCl) reference electrode (RE). This architecture was selected to evaluate the suitability of PLH as a substrate for printed electrochemical sensors under standard operating conditions.

**Figure 6:**
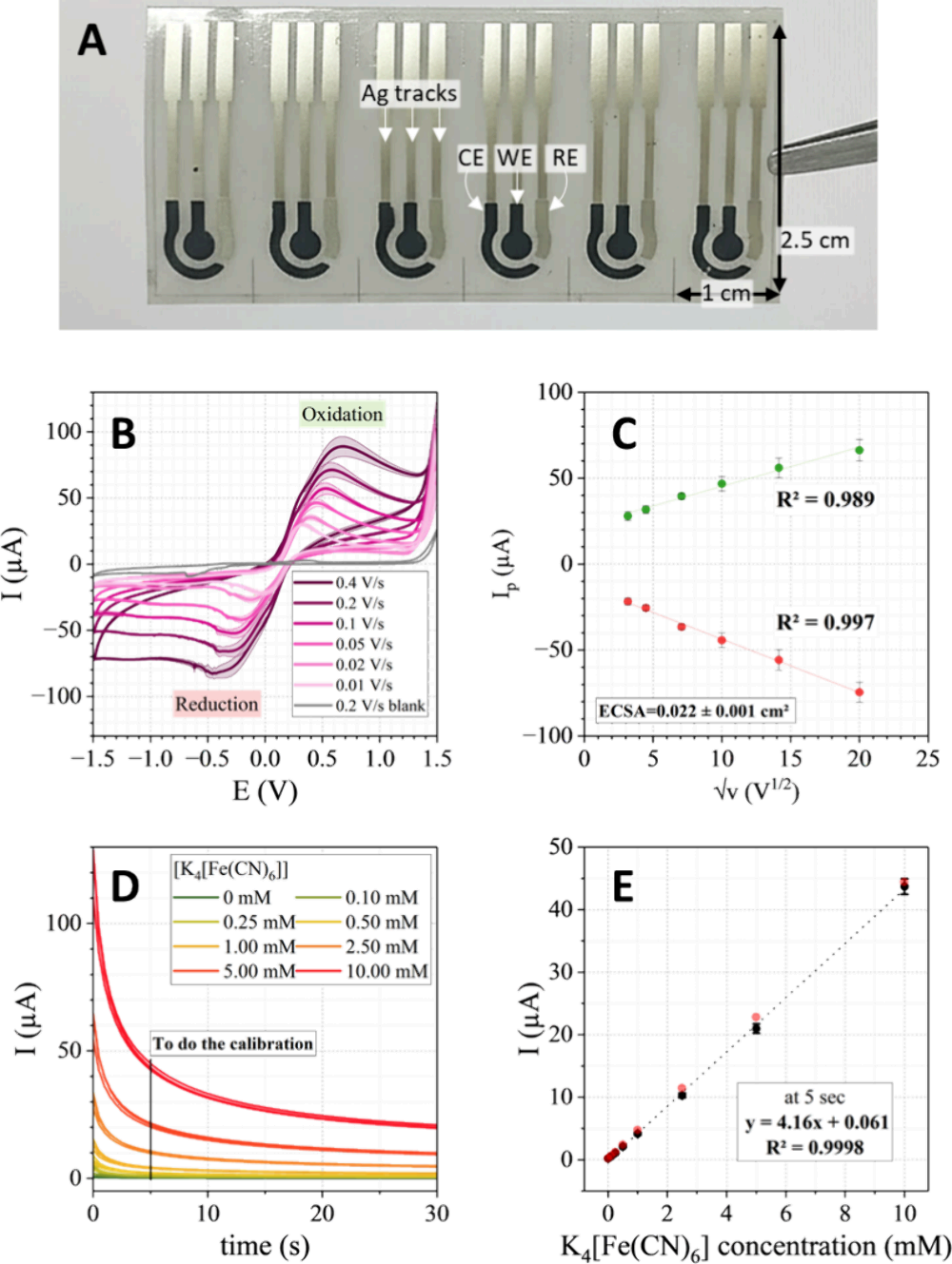
Development of an electrochemical sensor. (A) Printed three-electrode system on the PLH substrate. (B) Voltamograms recorded at different scan rates in 5 mM [Fe­(CN)_6_]^3‑/4‑^ and 0.1 M KCl; the inset shows the blank electrolyte without redox probe, confirming the electrochemical inertness of the PLH substrate. (C) Trumpet plot; linear dependence of the peak current on the square root of scan rate used to estimate the electrochemically active surface area (ECSA) according to Randles-Ševčík. (D) Chronoamperometry responses recorded at different [Fe­(CN)_6_]^4–^ concentrations applying 1.0 V vs Ag/AgCl. (E) Corresponding calibration plot showing a linear relationship between current intensity at 5 s and analyte concentration. Data points shown in red correspond to measurements performed 4 months after device fabrication.

The electrochemical performance of the printed device was first assessed by cyclic voltammetry (CV) using 5 mM potassium ferricyanide/ferrocyanide ([Fe­(CN)_6_]^3‑/4‑^) in 0.1 M KCl as a redox probe. CV were recorded at different scan rates (Figure 6B). Control experiments conducted in blank electrolyte (0.1 M KCl without redox species) showed no Faradaic peaks (inset of Figure 6B), indicating that no interference from PLH substrate is observed under tested conditions.

As shown in Figure 6C, both anodic and cathodic peak currents exhibit a linear dependence on the square root of the scan rate, consistent with a diffusion-controlled reversible redox process. This behavior confirms the electrochemical functionality and stability of the printed electrodes on the PLH substrate.

The electrochemical Active Surface Area (ECSA) of the printed working electrode was estimated using the Randles–Ševčík equation for a reversible redox system (eq 3):

$$I_{p}=\left(2.69\times 10^{5}\right)\cdot n^{3/2}\cdot A\cdot D^{1/2}\cdot C\cdot v^{1/2}$$ 3

where *I*_*p*_ is the peak current (A), *n* is the number of electrons transferred, *A* is the electroactive surface area (cm^2^), *D* is the diffusion coefficient of the redox species (cm^2^/s), *C* is the bulk concentration (mol/cm^3^), and *v* is the scan rate (V/s). Using the slope obtained from the linear fit of *I*_*p*_ versus *v* ^1/2^ and assuming a diffusion coefficient of 7.13 × 10^–6^ cm^2^/s for ferri-/ferrocyanide redox couple, an electrochemical active surface area (ECSA) of 0.022 ± 0.001 cm^2^ was determined. Considering the geometrical electrode area of 0.071 cm^2^, this corresponds to ∼ 32% utilization of the nominal surface area.

Subsequently, chronoamperometric measurements were performed at increasing ferricyanide concentrations in 0.1 M KCl by applying a constant potential of 1.0 V vs. Ag/AgCl (Figure 6D). The applied potential was selected based on the voltammetric characterization, as it lies beyond the anodic peak of the ferricyanide/ferrocyanide redox couple and ensures diffusion-limited oxidation under steady-state conditions. The corresponding calibration curve (Figure 6E) shows a linear relationship between the measured current (at 5 s) and analyte concentration, indicating good linearity, signal stability, and reproducibility of the printed sensor response. All electrochemical measurements were carried out using three independently fabricated devices, and the reported values correspond to the mean response (N = 3).

These results demonstrate that PLH substrates support the reliable fabrication of printed electrochemical sensors with stable and reproducible electrochemical behavior. Long-term measurements performed several months after fabrication are fully consistent with the initial calibration, with overlapping calibration data and no observable deviation in sensor response, indicating stable device performance over this time period. While detailed analytical optimization was beyond the scope of this study, the results confirm the suitability of PLH as a flexible and biobased substrate for printed electronic components.

## Conclusions

This work identifies poly­(lactic acid-*co*-oxacyclohexadecenlactone) (PLH) as a promising biobased substrate for flexible printed electronics. PLH provides wettability compatible with uniform ink deposition with good adhesion, and exhibits dielectric properties within the typical range of engineering polymers and high insulating strength. Compared to PET, PLH offers significantly higher flexibility while maintaining dimensional stability within the relevant processing window (<120 °C), enabling its use in applications requiring mechanical compliance without compromising printed features. Although its thermal stability is lower than PET, PLH remains compatible with moderate processing and operating temperatures typical of printed electronics.

Beyond material-level characterization, functionality was demonstrated through a proof-of-concept inductive sensor fabricated directly on the PLH substrates. The printed device exhibited stable impedance responses and distinct, reproducible phase shifts between liquids with different dielectric properties, confirming that PLH supports the fabrication and operation of active printed devices under realistic conditions.

Overall, the combination of printability, electrical insulation, mechanical compliance and biobased origin highlights the potential of PLH as an alternative to fossil-derived substrates for low-complexity flexible electronic applications. While PET benefits from established recycling infrastructures, its effective recyclability in printed electronic devices remains limited due to material integration. In this context, biobased polymers such as PLH offer complementary end-of-life strategies, particularly for short-lifecycle or disposable devices, where biodegradability may provide practical advantage.

Future work will focus on improving surface homogeneity, enabling double-sided printing, enhancing thermal stability, and evaluating long-term mechanical durability under cyclic deformation to further advance PLH toward practical device integration.

## Supplementary Material



## Notes

Figure S1, experimental procedure used to evaluate the thermomechanical and tensile behavior of PLH substrates, schematic representations of the sample layout, photographs of thermally treated specimens, and images of the custom tensile testing setup along with representative results after mechanical testing (PDF)
