# Supplementary material for: Poly(lactic acid-co-oxacyclohexadecenlactone) (PLH): A Bio-Based Substrate for Flexible Printed Electronic Devices
Source: ACS Appl Mater Interfaces. 2026 Jun 3;18(23):33192–200. doi: 10.1021/acsami.6c04927 (PMC13288377; doi:10.1021/acsami.6c04927)
Supplement: Supplementary file 1 [file am6c04927_si_001.pdf]

<u>Supplementary Information:</u>

Poly(lactic acid-co-oxacyclohexadecenlactone) (PLH): A Bio-Based Substrate for Flexible Printed Electronic Devices

AUTHOR NAMES

Aida Visús Martínez,[a] Nathalie Marcela Cerón[b] José L. Monzón,[b] Miquel Redón,[a] David Sánchez,[a] Jordi Sacristán,[a] Xavier Jordà,[a] Carlos Domínguez Horna*[a], Francesc Xavier Muñoz Berbel*[a]*

AUTHOR ADDRESS

[a] Institute of Microelectronics of Barcelona (IMB-CNM), Universitat Autònoma de Barcelona, Campus UAB, 08193 Bellaterra, Spain

[b] Artificial Nature S.L., Carrer de Baldiri Reixac 10, 08028 Barcelona, Spain

*Corresponding authors. Email: n.marcela@artificialnature.com;

carlos.dominguez@imb-cnm.csic.es; Xavier.Munoz@imb-cnm.csic.es

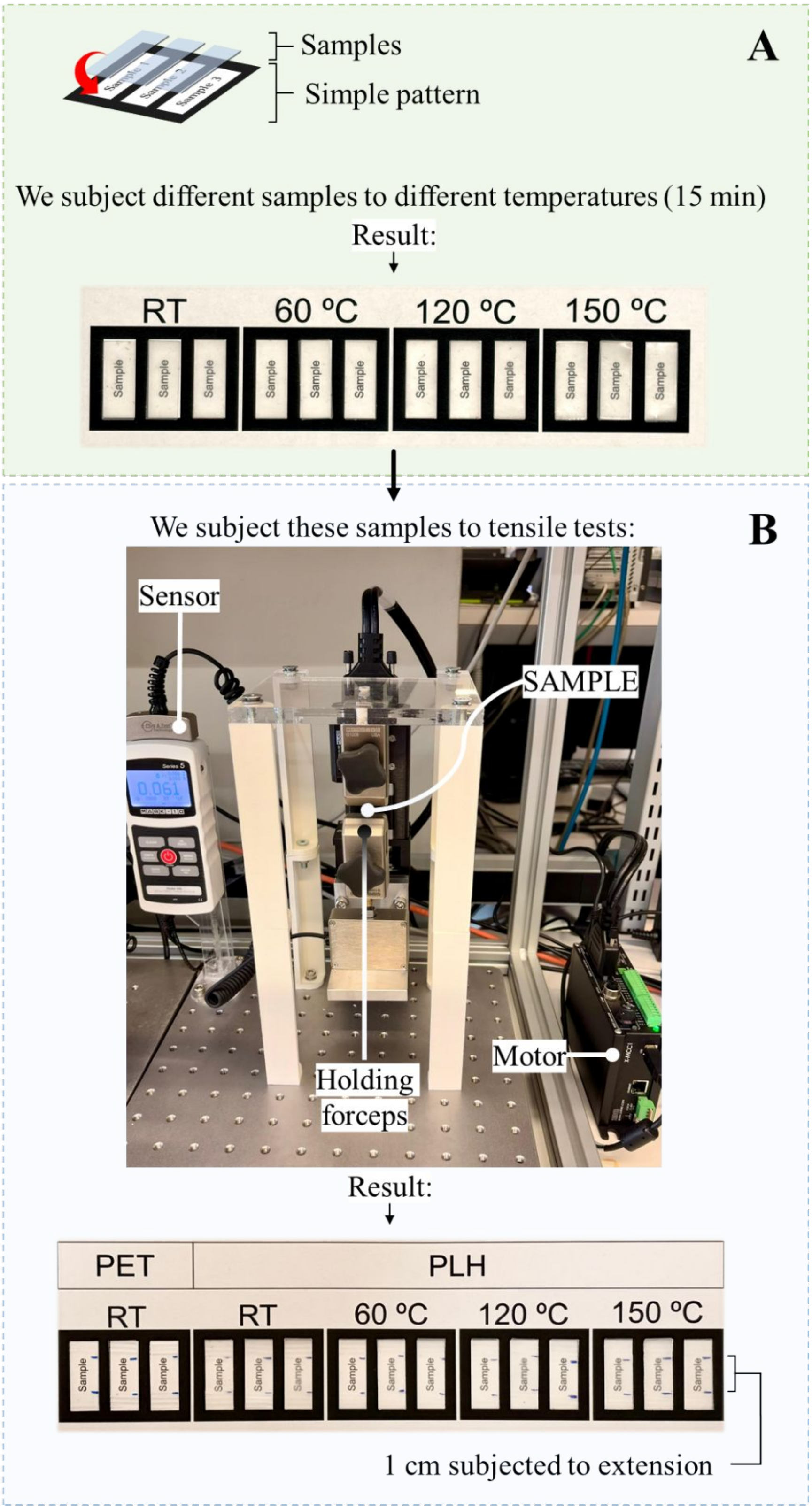

Figure S1. Experimental procedure used to evaluate the thermo-mechanical and tensile behavior of PLH substrates. (A) Schematic representation of the sample layout and photographs of PLH specimens after thermal treatment at room temperature (RT), 60 °C, 120 °C, and 150 °C for 15 min, used to assess macroscopic dimensional stability and potential thermal expansion or contraction. (B) Photograph of the custom tensile testing setup, showing the force sensor, holding forceps, motor, and sample positioning. Representative images of PET and PLH samples after tensile testing are shown below, corresponding to an applied extension of 1 cm under the indicated thermal treatment conditions.
